# Birdwatching as a Nature-Based Behavioral Strategy to Improve Adherence to Pelvic Floor Muscle Training in Women With Stress Urinary Incontinence: A Prospective Comparative Observational Study

**DOI:** 10.7759/cureus.112975

**Published:** 2026-07-19

**Authors:** Nobuo Okui, Machiko Okui

**Affiliations:** 1 Urogynecology, Yokosuka Urogynecology and Urology Clinic, Yokosuka, JPN; 2 Mathematics, Kanagawa Dental University, Yokosuka, JPN

**Keywords:** birdwatching, gamification, pelvic floor muscle training, stress urinary incontinence, women's health

## Abstract

Background

Long-term adherence remains one of the greatest barriers to successful pelvic floor muscle training (PFMT) for women with stress urinary incontinence (SUI). Although digital gamification strategies have been developed to improve adherence, they often require electronic devices and digital literacy. We evaluated whether birdwatching, as a simple nature-based behavioral strategy designed to promote motivation through environmental cueing and habit formation, was associated with improved adherence to PFMT and better clinical outcomes in women with SUI.

Methods

This prospective comparative observational study included women with SUI who received standardized supervised PFMT. After receiving an explanation of both management strategies, participants chose either conventional PFMT alone or PFMT combined with a birdwatching-based behavioral strategy according to their preference. The primary outcome was overall PFMT adherence during the 12-week follow-up, calculated using weather-adjusted eligible walking days. Secondary outcomes included the one-hour pad test, SUI episode frequency, Patient Global Impression of Improvement (PGI-I), and Brink scale. Clinical outcomes were evaluated after 12 weeks.

Results

After propensity score matching, 48 women were included in the final analysis (standard PFMT, *n* = 24; birdwatching, *n* = 24). Although overall PFMT adherence gradually declined in both groups, the birdwatching group demonstrated significantly higher overall PFMT adherence at Week 12. Compared with the standard PFMT group, participants in the birdwatching group also experienced significantly fewer SUI episodes. No significant between-group differences were observed in the one-hour pad test, PGI-I score, or Brink scale score. No intervention-related adverse events were observed.

Conclusions

A simple birdwatching-based behavioral strategy was associated with improved adherence to PFMT and favorable short-term clinical outcomes compared with standard PFMT alone. Unlike previously reported digital interventions, this nature-based approach requires no electronic devices or specialized technology and may represent a practical, inexpensive, and scalable behavioral strategy for improving long-term adherence to conservative treatment for women with SUI.

## Introduction

Stress urinary incontinence (SUI) is one of the most common pelvic floor disorders affecting women and substantially impairs quality of life, physical activity, and social participation [1,2]. Current international guidelines recommend pelvic floor muscle training (PFMT) as the first-line conservative treatment because it improves pelvic floor muscle strength, reduces urinary leakage, and avoids the risks associated with surgical intervention [3,4]. Numerous randomized controlled trials and systematic reviews have demonstrated the clinical efficacy of PFMT for women with SUI [5,6].

However, the effectiveness of PFMT in everyday clinical practice depends not only on the exercise itself but also on whether patients continue performing it [7,8]. Poor long-term adherence remains the principal barrier to successful PFMT. Although many women initially perform the prescribed exercises, adherence commonly declines over time because repetitive home-based training is monotonous, lacks immediate feedback, and is often perceived as an obligation rather than an enjoyable daily habit [9]. Previous clinical studies have consistently shown that women who perform PFMT more regularly achieve better continence outcomes than those with poor adherence, highlighting sustained exercise behavior as a major determinant of treatment success [10,11].

Improving long-term adherence has therefore become a major focus of behavioral intervention research [12,13]. Behavioral strategies designed to promote motivation and support habit formation have emerged as promising approaches for sustaining health-related behaviors. Rather than relying solely on scheduled reminders or external reinforcement, some interventions encourage active engagement with the surrounding environment, allowing naturally occurring cues to become incorporated into everyday routines. Such approaches may enhance motivation by combining voluntary exploration with the anticipation of unpredictable and rewarding experiences, thereby facilitating long-term adherence. Many interventions developed for PFMT and other rehabilitation programs have implemented these behavioral principles through digital technologies, including smartphone applications, wearable sensors, reminder systems, and virtual or augmented reality [14,15]. Although these interventions can improve engagement, they also depend on electronic devices, software maintenance, and varying levels of digital literacy, potentially limiting their accessibility, particularly among middle-aged and older women [16].

Nature-based behavioral interventions have recently emerged as a promising alternative for promoting health-related behaviors without relying on digital technology [17,18]. Walking is already widely recommended as a healthy daily activity, and exposure to natural environments during walking has been associated with improvements in both physical and mental health [18,19]. Birdwatching naturally encourages people to remain attentive to their surroundings while spending time outdoors. Associating pelvic floor muscle contractions with bird observations during routine walking may transform repetitive exercise into an enjoyable experience supported by naturally occurring environmental cues rather than scheduled reminders. Because this approach requires no electronic devices, specialized equipment, or technical skills, it represents a simple, low-cost, and potentially scalable nature-based behavioral strategy designed to promote motivation through environmental cueing and habit formation.

Despite growing interest in behavioral strategies for improving adherence and in nature-based health interventions, birdwatching has not previously been evaluated as a strategy for improving adherence to PFMT in women with SUI. In this pilot prospective comparative study, we investigated whether a birdwatching-based behavioral strategy could improve adherence to PFMT compared with standard PFMT instruction. We also evaluated whether improved adherence was associated with better clinical outcomes, including urinary incontinence severity, patient-reported outcomes, and pelvic floor muscle strength after 12 weeks.

## Materials and methods

Study design and participants

This prospective observational comparative pilot study was conducted at the Yokosuka Urogynecology and Urology Clinic (Yokosuka, Kanagawa, Japan) between October 2025 and March 2026. Women presenting to the outpatient clinic with SUI were consecutively enrolled. Eligible participants had SUI symptoms, a positive one-hour pad test, the ability to walk independently outdoors, and the ability to complete daily walking and PFMT diaries. Exclusion criteria included urgency urinary incontinence as the predominant symptom, urinary tract infection, neurological disorders affecting lower urinary tract function, severe pelvic organ prolapse, inability to voluntarily contract the pelvic floor muscles, or inability to complete the scheduled follow-up. The study protocol was approved by the Ethics Committee of Yokosuka Urogynecology and Urology Clinic (Approval No. 2025Jun001). Written informed consent was obtained from all participants before enrollment.

Group allocation

After receiving standardized education regarding conventional PFMT and the optional birdwatching-based behavioral strategy, all participants were informed about both approaches. Participants then selected their preferred management strategy according to their own preference. Women who chose conventional PFMT comprised the standard PFMT group, whereas those who chose to incorporate birdwatching into their routine walking comprised the birdwatching group. Both groups received identical conventional PFMT education. The only difference between groups was the behavioral strategy used to promote long-term adherence.

Standard PFMT program

All participants received standardized education regarding PFMT from a urogynecologist during pelvic examination. Correct pelvic floor muscle contraction was demonstrated using transperineal ultrasonography so that patients could visually confirm muscle movement in real time. A QR code linking to an instructional YouTube video demonstrating the exercise technique was provided as a reference.

Pelvic floor muscle strength was assessed using the Brink scale [20]. Two independent physicians evaluated each participant separately, and the average Brink score was used for analysis.

Birdwatching-based behavioral strategy

Participants in the birdwatching group additionally received a brief explanation from the physician regarding local birdwatching and were encouraged to associate bird observation with PFMT. Instead of performing exercises only according to obligation or schedule, participants were instructed to perform at least one PFMT session whenever they observed a bird during walking. This intervention was designed as a simple, low-cost, non-digital behavioral strategy using naturally occurring environmental cues to support motivation and habit formation.

Participants were encouraged to walk on one of four approximately one-km flat walking courses located in Yokosuka City.

Walking diary and follow-up

Participants in both groups recorded daily walking activity and PFMT performance in a paper diary. Follow-up assessments were performed at Weeks 4, 8, and 12.

Walking adherence (%) was defined as the percentage of eligible walking days on which walking was completed.

PFMT adherence during walking (%) was defined as the percentage of walking days on which PFMT was performed.

Overall PFMT adherence (%) was calculated as:

Walking adherence (%) × PFMT adherence during walking (%) / 100

Because walking depended on weather conditions, adherence calculations used the number of eligible walking days as the denominator rather than total calendar days. During the study period, the numbers of eligible walking days were 22 in October 2025, 23 in November, 24 in December, 25 in January 2026, 21 in February, and 18 in March, reflecting local weather conditions. Representative walking routes and examples of the paper diary forms used in the study are provided in the Appendices.

Follow-up

Participants were followed for 12 weeks, with study assessments performed at Weeks 4, 8, and 12. Participants who completed follow-up underwent propensity score matching before the final comparative analysis.

Statistical analysis

Continuous variables are presented as mean ± standard deviation (SD) or median with interquartile range (IQR), according to data distribution, whereas categorical variables are presented as frequencies and percentages. Baseline characteristics and continuous clinical outcomes were compared using Welch's t-test, and categorical variables were compared using Fisher's exact test. Overall PFMT adherence at Weeks 4, 8, and 12 was compared between groups using Welch's t-test.

Propensity scores were estimated by logistic regression using the following baseline covariates: age, body mass index, parity, anterior compartment stage, posterior compartment stage, apical compartment stage, one-hour pad test result (g), daily SUI episode frequency, and Brink scale score. One-to-one nearest-neighbor propensity score matching without replacement was performed using a caliper width of 0.2 standard deviations of the logit of the propensity score. The propensity score-matched cohort was used for all comparative analyses.

Test statistics (t values) are reported alongside corresponding p values for continuous variables. Two-sided p-values < 0.05 were considered statistically significant. Statistical analyses were performed using IBM SPSS Statistics for Windows, Version 31 (Released 2025; IBM Corp., Armonk, New York, United States).

## Results

Study population

**Figure 1:**
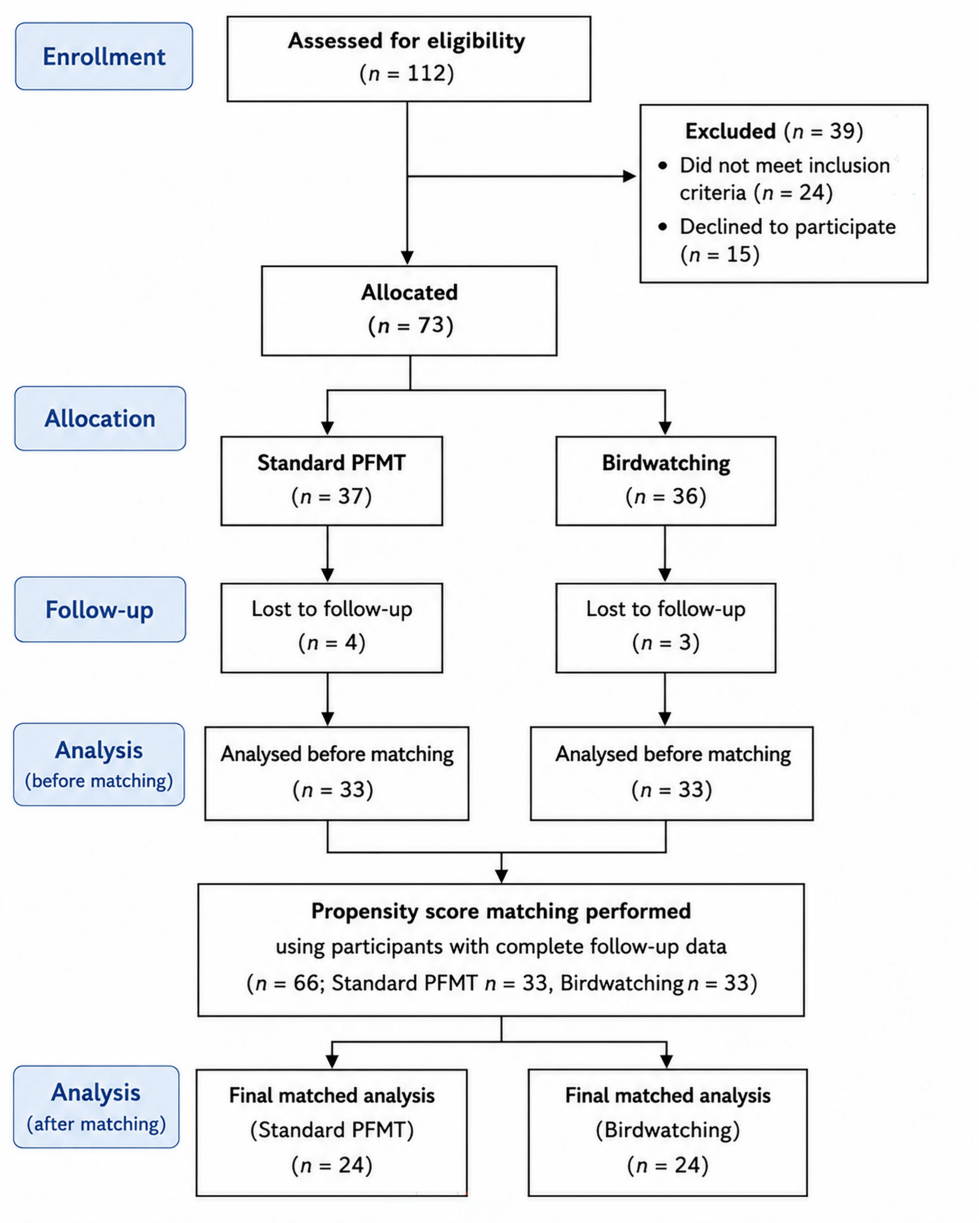
Participant flow diagram of the study Assessment for eligibility, enrollment, participant choice, follow-up, propensity score matching, and final analysis. PFMT, pelvic floor muscle training.

Figure 1 illustrates the participant flow from recruitment to the final propensity score-matched analysis. Between October 2025 and March 2026, 112 women with SUI were assessed for eligibility. Of these, 39 were excluded, including 24 who did not meet the inclusion criteria and 15 who declined to participate. The remaining 73 participants chose either the standard PFMT program (n = 37) or the birdwatching program (n = 36) according to their preference.

During the 12-week follow-up, four participants in the standard PFMT group and three participants in the birdwatching group were lost to follow-up, leaving 33 participants in each group who completed the study. Propensity score matching was subsequently performed immediately before the final statistical analysis using the baseline characteristics of these 66 participants. One-to-one nearest-neighbor matching with a caliper width of 0.2 standard deviations of the logit of the propensity score identified 24 matched pairs (24 participants per group), which constituted the final matched cohort for all comparative analyses.

Baseline characteristics

Table 1 summarizes the baseline characteristics of the study participants. No statistically significant differences were observed between the standard PFMT group and the birdwatching group with respect to age (t = 0.37, p = 0.712), body mass index (t = −1.59, p = 0.119), parity (t = 0.86, p = 0.397), anterior, posterior, or apical compartment stage (all Fisher's exact test, p ≥ 1.000), 1-hour pad test results (t = −0.09, p = 0.925), SUI episodes (t = 0.34, p = 0.738), and Brink scale scores (t = 0.00, p = 1.000), indicating comparable baseline characteristics between the two groups.

**Table 1:** Baseline characteristics of the study participants Values as mean ± standard deviation, median [interquartile range], or n (%). Welch's t-test (continuous variables), Fisher's exact test (categorical variables), Mann–Whitney U test (Brink scale), standardized mean differences (SMDs) for post-match covariate balance, and N/A for Fisher's exact test statistics. BMI, body mass index; POP, pelvic organ prolapse; SUI, stress urinary incontinence; PFMT, pelvic floor muscle training.

Variable	Standard PFMT (n = 24)	Birdwatching (n = 24)	Test statistic	p-value	SMD
Age (years)	59.3 ± 4.9	58.7 ± 6.0	t = 0.37	0.712	0.11
BMI (kg/m²)	26.5 ± 2.3	27.8 ± 3.3	t = −1.59	0.119	0.46
Parity	2.21 ± 0.59	2.08 ± 0.41	t = 0.86	0.397	0.25
Anterior compartment, n (%)	19 (79.2%)	18 (75.0%)	N/A	1.000	0.10
Posterior compartment, n (%)	13 (54.2%)	13 (54.2%)	N/A	1.000	0.00
Apical compartment, n (%)	20 (83.3%)	19 (79.2%)	N/A	1.000	0.11
1-hour pad test (g)	3.01 ± 1.05	3.03 ± 0.76	t = −0.09	0.925	0.03
SUI episodes/day	1.79 ± 0.41	1.75 ± 0.44	t = 0.34	0.738	0.10
Brink scale	6 [6–6]	6 [6–6]	U = 288.0	1.000	0.00

Clinical outcomes at Week 12

Table 2 summarizes the clinical outcomes at the 12-week follow-up and the changes from baseline. At Week 12, the birdwatching group experienced significantly fewer SUI episodes than the standard PFMT group (t = 3.22, p = 0.002). No significant between-group differences were observed in the one-hour pad test (t = 1.58, p = 0.120), SUI cure rate (Fisher's exact test, p = 0.365), PGI-I score [21] (t = 1.92, p = 0.061), or Brink scale score (t = −0.90, p = 0.371).

**Table 2:** Clinical outcomes at the 12-week follow-up and changes from baseline Values as mean ± standard deviation, median [interquartile range], or n (%). Welch's t-test for continuous variables, Fisher's exact test for SUI cure, Mann–Whitney U test for between-group comparisons of Brink scale scores, Wilcoxon signed-rank test for within-group changes in Brink scale, and change from baseline for within-group differences. N/A, not applicable. PFMT, pelvic floor muscle training; SUI, stress urinary incontinence; PGI-I, Patient Global Impression of Improvement; SD, standard deviation.

Variable	Standard PFMT	Birdwatching	Test statistic	p-value	Change from baseline (Standard PFMT)	Change from baseline (Birdwatching)	Test statistic	p-value
1-hour pad test (g)	2.27 ± 1.69	1.51 ± 1.62	t = 1.58	0.120	−0.74 ± 0.91	−1.53 ± 1.94	t = 1.77	0.083
SUI cure, n (%)	6 (25.0%)	9 (37.5%)	N/A	N/A	N/A	N/A	N/A	N/A
SUI episodes/day	1.38 ± 0.88	0.58 ± 0.83	t = 3.22	0.002	−0.42 ± 0.78	−1.17 ± 0.96	t = 2.96	0.005
PGI-I	3.04 ± 1.33	2.29 ± 1.37	t = 1.92	0.061	N/A	N/A	N/A	N/A
Brink scale	7 [6–8]	8 [7–9]	U = 248.0	0.385	+1 [0–2]	+1 [1–2]	W = 0.0 / W = 7.0	0.011/0.001

Analysis of changes from baseline showed significantly greater reductions in SUI episodes in the birdwatching group than in the standard PFMT group (t = 2.96, p = 0.005). Although greater improvement in the 1-hour pad test was also observed in the birdwatching group, the between-group difference did not reach statistical significance (t = 1.77, p = 0.083). Likewise, changes in Brink scale scores were comparable between the two groups (t = −0.91, p = 0.365).

Overall PFMT adherence

**Figure 2:**
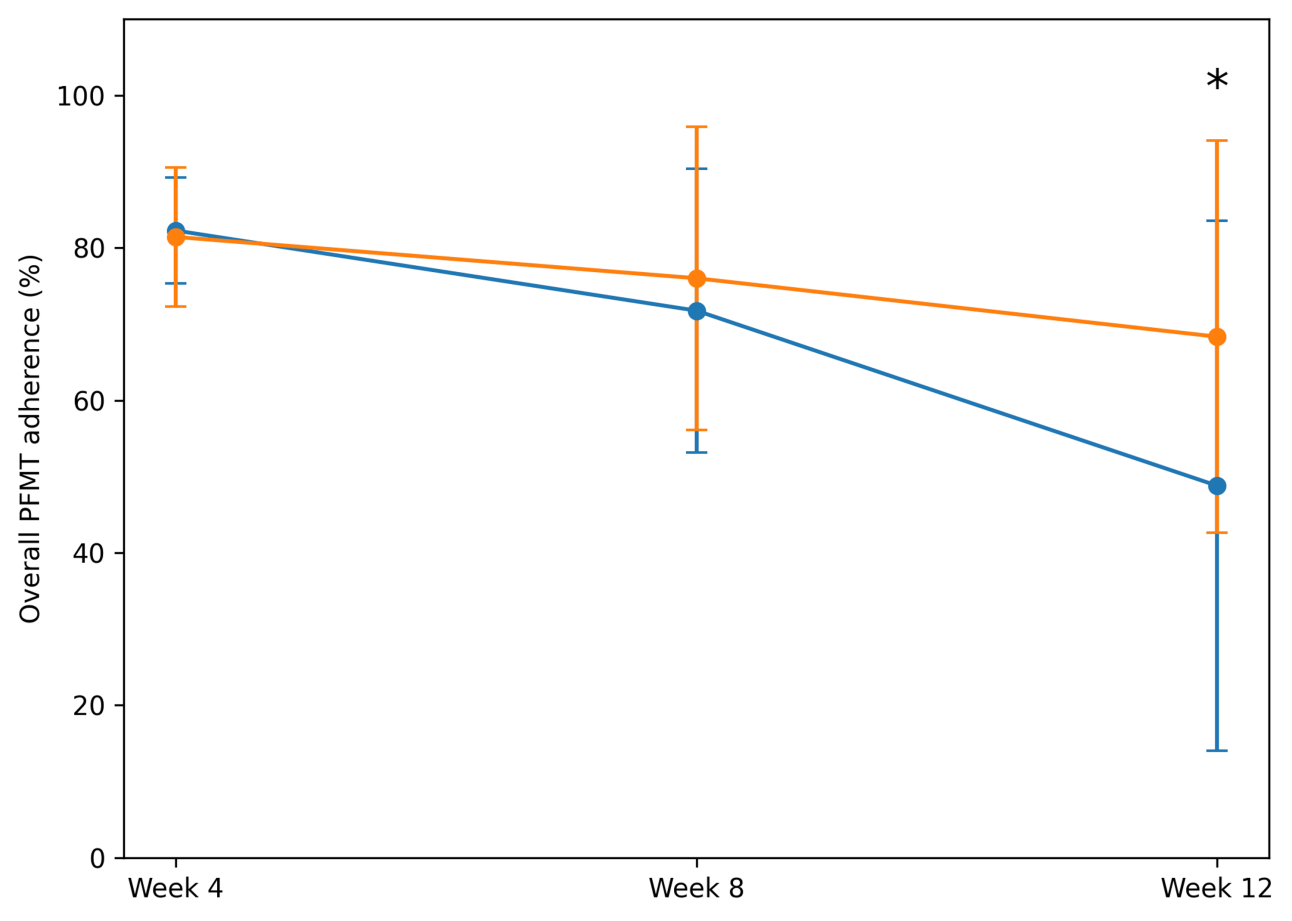
Longitudinal changes in overall pelvic floor muscle training adherence during the 12-week intervention Overall pelvic floor muscle training adherence in the standard pelvic floor muscle training group and the birdwatching group at Weeks 4, 8, and 12. Overall PFMT adherence, calculated as the product of walking adherence and PFMT adherence during walking. Data presented as mean ± standard deviation. PFMT, pelvic floor muscle training. p < 0.05 versus the standard PFMT group at the corresponding time point (Week 12; Welch's t-test, t = −2.22, p = 0.032). PFMT, pelvic floor muscle training

Figure 2 shows the longitudinal changes in overall PFMT adherence during the 12-week intervention. Overall PFMT adherence gradually declined over time in both groups. No significant between-group differences were observed at Week 4 (t = 0.36, p = 0.723) or Week 8 (t = −0.76, p = 0.449). However, at Week 12, the birdwatching gamification group maintained significantly higher overall PFMT adherence than the standard PFMT group (68.3% vs. 48.8%; t = −2.22, p = 0.032).

Overall PFMT adherence

Table 3 summarizes overall PFMT adherence during the 12-week follow-up. Overall PFMT adherence gradually declined over time in both groups. No significant between-group differences were observed at Week 4 (t = 0.36, p = 0.723) or Week 8 (t = −0.76, p = 0.449). However, by Week 12, the birdwatching group maintained significantly higher overall PFMT adherence than the standard PFMT group (68.3% ± 25.7% vs. 48.8% ± 34.7%; t = −2.22, p = 0.032).

**Table 3:** Overall pelvic floor muscle training adherence during the 12-week follow-up Overall pelvic floor muscle training adherence at Weeks 4, 8, and 12 in the standard pelvic floor muscle training and birdwatching groups. Values are presented as mean ± standard deviation (%). Overall PFMT adherence (%) was calculated as walking adherence (%) × PFMT adherence during walking (%) / 100. Between-group comparisons were performed using Welch's t-test. PFMT, pelvic floor muscle training; SD, standard deviation.

Time point	Standard PFMT (n = 24)	Birdwatching (n = 24)	Test statistic	p-value
Week 4	82.2 ± 6.9	81.4 ± 9.1	t = 0.36	0.723
Week 8	71.8 ± 18.6	76.0 ± 19.9	t = −0.76	0.449
Week 12	48.8 ± 34.7	68.3 ± 25.7	t = −2.22	0.032

## Discussion

This pilot prospective comparative study demonstrated that a simple birdwatching-based gamification strategy improved long-term adherence to PFMT in women with SUI. Although adherence gradually declined in both groups during the 12-week follow-up, participants in the birdwatching group consistently maintained higher adherence and demonstrated significantly greater overall PFMT adherence at Week 12. This behavioral improvement was accompanied by greater reductions in urinary leakage, fewer SUI episodes, and larger improvements in disease-specific quality of life. Because both groups received identical PFMT instruction and differed only in the behavioral strategy used to encourage exercise continuation, these findings suggest that improved adherence contributed to the observed clinical benefits.

These findings are consistent with those of a recent randomized controlled trial evaluating a mobile application for PFMT in women with SUI. In that study, participants using the mobile application maintained significantly higher PFMT adherence during the 12-week intervention and showed greater improvements in symptom-related quality of life than those receiving standard instruction, although objective continence outcomes were similar between groups [22]. Similarly, our birdwatching-based behavioral strategy was associated with higher PFMT adherence over the same follow-up period and with better clinical outcomes. Unlike the mobile application, however, our intervention required no smartphone, reminder system, or electronic device, instead using naturally occurring environmental cues during routine walking to encourage regular PFMT.

The importance of adherence has been consistently emphasized in previous PFMT research. International guidelines recommend supervised PFMT as the first-line conservative treatment for SUI, and systematic reviews have demonstrated that PFMT improves urinary incontinence outcomes in women with SUI [5,6]. However, maintaining long-term exercise behavior remains one of the greatest challenges in routine clinical practice. Many patients discontinue regular PFMT practice because the training becomes repetitive, lacks immediate reward, and is difficult to incorporate into everyday life [9]. Previous studies have also shown that women who perform PFMT more regularly achieve better continence outcomes than those with poor adherence [11]. Our findings support the concept that improving adherence may be as important as optimizing the exercise program itself. This interpretation is consistent with the International Continence Society consensus statement, which identified the development of effective strategies to improve long-term PFMT adherence as a major priority for future research [23]. Likewise, a recent systematic review and meta-analysis reported that higher adherence to pelvic floor muscle exercise therapy was associated with significantly greater improvements in quality of life, while also emphasizing the need for more standardized methods of measuring adherence [24].

A distinctive feature of the present study is that adherence was promoted using a nature-based behavioral strategy rather than a technology-dependent behavioral intervention. Most behavioral interventions developed to improve PFMT adherence have relied on smartphone applications and other digital health technologies to encourage regular exercise [12-15]. Although these approaches can improve motivation and adherence, they also require electronic devices, software maintenance, and varying levels of digital literacy [13,16]. In contrast, our strategy used birdwatching to promote active engagement with the surrounding environment during routine walking. Rather than relying on reminders or applications, participants were encouraged to associate bird observations with PFMT, allowing naturally occurring environmental cues to support motivation and habit formation. This behavioral association may explain the consistently higher adherence observed throughout follow-up. Unlike previous digital behavioral interventions, our strategy requires no electronic devices or specialized equipment and may provide a practical, inexpensive, and scalable alternative for women who have limited access to or preference for digital health technologies, particularly in community and primary care settings [17,22].

The mechanism underlying this intervention is likely behavioral rather than physiological. Birdwatching does not directly strengthen the pelvic floor muscles. Instead, it provides a motivating behavioral context that encourages repeated PFMT practice through active engagement with the surrounding environment. Previous studies have demonstrated that everyday encounters with birdlife are associated with sustained improvements in mental well-being, and that even brief birdwatching activities can enhance well-being, reduce anxiety, and strengthen nature connectedness [24,25]. Furthermore, a recent experimental study reported that repeated birdwatching sessions increased psychological well-being and reduced psychological distress compared with a control condition [26]. These positive emotional experiences, together with naturally occurring environmental cues, may reinforce repeated PFMT practice and facilitate habit formation, allowing pelvic floor exercises to become integrated into routine walking rather than remaining an isolated therapeutic task. Consistent with these findings, our study extends the potential benefits of birdwatching beyond psychological well-being by suggesting that a nature-based behavioral strategy incorporating environmental cueing and habit formation may also promote long-term adherence to a therapeutic exercise program.

From a clinical perspective, this intervention offers several practical advantages. It is inexpensive, requires no electronic devices, internet access, or software, and can easily be incorporated into routine outpatient care with minimal additional instruction. Because walking is already recommended as a healthy lifestyle activity, combining walking with birdwatching and PFMT represents a feasible strategy that could be implemented in community-based rehabilitation and primary care settings, particularly for middle-aged and older women who may be less familiar with digital health technologies. Unlike previously reported smartphone- or application-based interventions, which depend on digital platforms to support adherence [22], the present behavioral strategy integrates PFMT into a healthy daily activity by using naturally occurring environmental cues to promote motivation and habit formation, potentially making it more accessible in routine clinical practice.

The present intervention differs from reminder-based behavioral approaches because participants actively engaged with the surrounding natural environment while walking. Birdwatching involves active observation and unpredictable encounters with birdlife, both of which may help sustain voluntary participation through intrinsic motivation and curiosity [27,28]. Although these behavioral mechanisms were not directly evaluated, they may partly explain the higher adherence observed in the birdwatching group.

Several limitations should be acknowledged. First, this was a single-center pilot study with a relatively small sample size, limiting statistical power and generalizability. Second, this was not a randomized controlled trial. Participants selected their preferred intervention rather than being randomly assigned, and residual confounding cannot be completely excluded despite propensity score matching. Although post-matching balance was substantially improved, a small residual imbalance remained for baseline BMI and parity. Notably, the birdwatching group had a slightly higher BMI, a factor generally associated with worse SUI outcomes. Therefore, this residual imbalance would be expected to bias the results against, rather than in favor of, the intervention. In addition, participants who chose the birdwatching intervention may have been more likely to engage with the intervention because of their personal preferences or motivation, introducing potential self-selection bias. Third, follow-up was limited to 12 weeks, and the long-term sustainability of the observed behavioral changes remains unknown. Fourth, adherence was assessed using self-reported paper diaries, which may have introduced reporting bias. Finally, only one nature-based behavioral strategy was evaluated. Future multicenter randomized controlled trials with longer follow-up are needed to determine whether similar adherence benefits can be achieved using birdwatching in other populations or through alternative nature-based environmental cues.

## Conclusions

The birdwatching-based behavioral strategy was associated with higher adherence to PFMT and with more favorable short-term urinary incontinence outcomes than standard PFMT instruction alone. These findings suggest that simple, low-cost, non-digital nature-based behavioral strategies may represent a practical approach to enhancing adherence to conservative treatment for women with SUI.

## Figures and Tables

**Figure 3:**
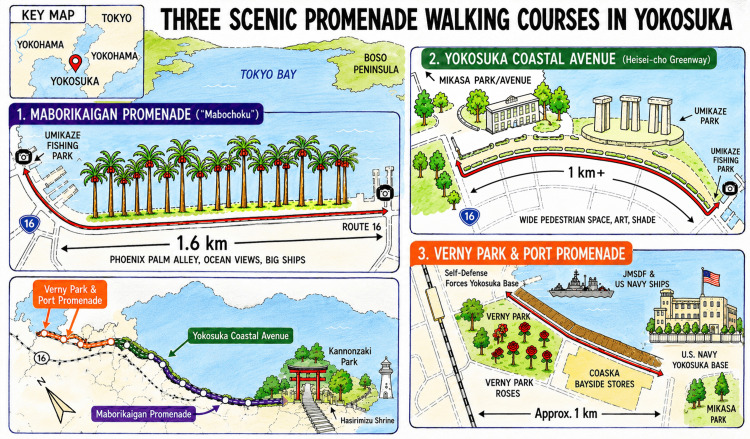
Representative walking routes used for the birdwatching intervention Three recommended walking routes in Yokosuka City, Japan, for the birdwatching intervention: Maborikaigan Promenade, Yokosuka Coastal Avenue, and Verny Park & Port Promenade. Flat, barrier-free promenades without steep slopes, suitable for middle-aged and older women. Safe walking environments with abundant wild bird populations throughout the year, facilitating the integration of birdwatching with pelvic floor muscle training during routine walking. Illustration was drawn by Nobuo Okui on an iPad using an Apple Pencil and subsequently colored and completed using Adobe Illustrator (version 30.6). Figure layout was edited using Adobe Photoshop (version 27.8) (Adobe Inc., San Jose, California, USA).

**Figure 4:**
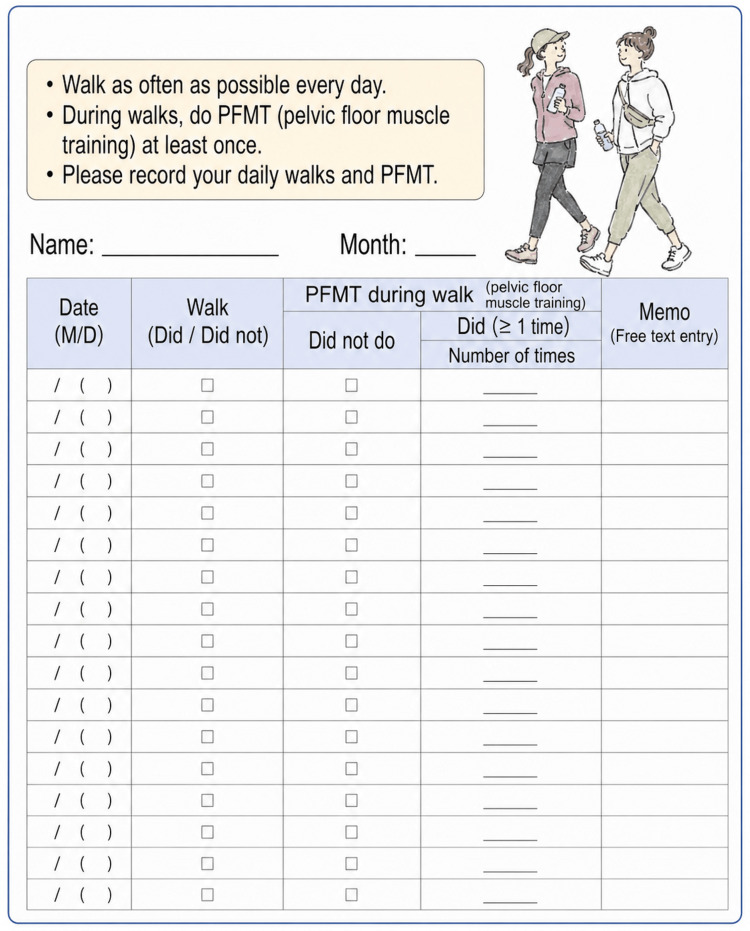
Daily walking and PFMT recording sheet Daily recording sheet for walking and pelvic floor muscle training (PFMT). Sections for daily walking, PFMT performed during walking, number of PFMT sessions, and free-text comments. Source document originally developed in Japanese and translated into English for publication. Illustration was drawn by Nobuo Okui on an iPad using an Apple Pencil and subsequently colored and completed using Adobe Illustrator (version 30.6). Figure layout was edited using Adobe Photoshop (version 27.8) (Adobe Inc., San Jose, California, USA).

**Figure 5:**
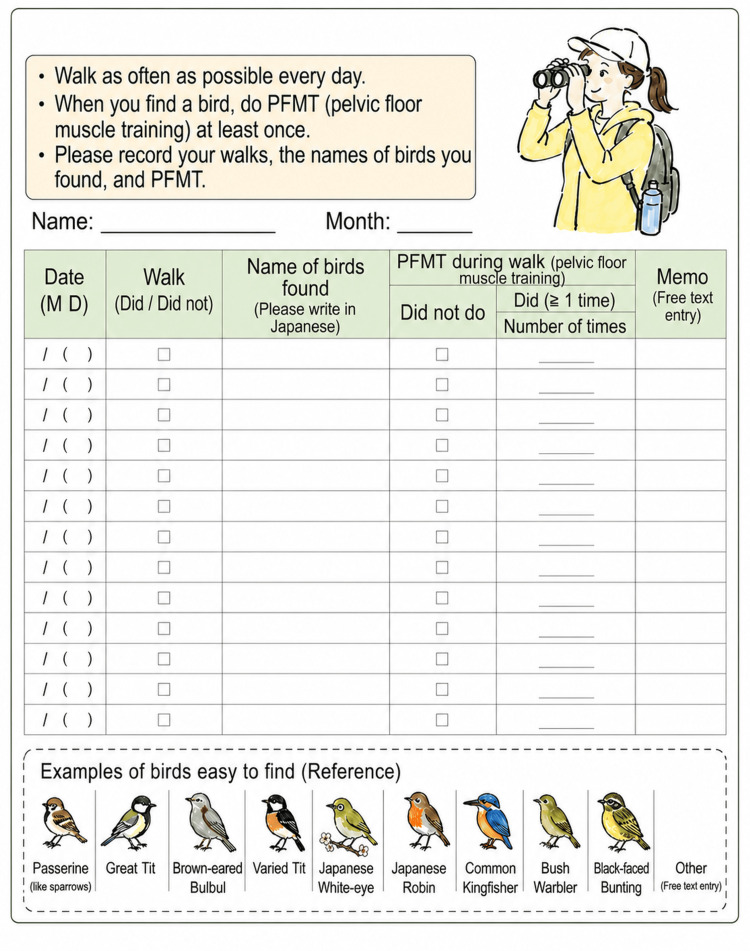
Daily walking and birdwatching-PFMT recording sheet Daily recording sheet for walking, birdwatching, and pelvic floor muscle training (PFMT). Sections for daily walking, names of birds observed, PFMT performed during walking, number of PFMT sessions, free-text comments, and illustrations of representative local bird species as visual references. Original document in Japanese, translated into English for publication. Illustration was drawn by Nobuo Okui on an iPad using an Apple Pencil and subsequently colored and completed using Adobe Illustrator (version 30.6). Figure layout was edited using Adobe Photoshop (version 27.8) (Adobe Inc., San Jose, California, USA).

## Appendices

Representative walking routes used in the study

Figure 3 shows the three recommended walking routes for the birdwatching intervention in Yokosuka City, Japan. The routes comprise flat, barrier-free promenades without steep slopes, providing safe and accessible walking environments for middle-aged and older women. Their abundant wild bird populations offer natural opportunities to combine birdwatching with pelvic floor muscle training during routine walking.

Daily walking and PFMT recording sheet

Figure 4 shows the daily walking and PFMT recording sheet used in the standard PFMT group. Participants were instructed to walk as often as possible, perform PFMT at least once during each walk, and record their daily walking and PFMT practice throughout the intervention period. The diary was used to calculate walking adherence, PFMT adherence during walking, and overall PFMT adherence.

Figure 5 shows the daily recording sheet used in the birdwatching group. In addition to recording daily walking and PFMT, participants recorded the names of birds observed during each walk. The sheet also included illustrations of common local bird species as visual references to encourage bird identification and reinforce the association between birdwatching and PFMT practice during routine walking.
